# Curvature Continuous and Bounded Path Planning for Fixed-Wing UAVs

**DOI:** 10.3390/s17092155

**Published:** 2017-09-19

**Authors:** Xiaoliang Wang, Peng Jiang, Deshi Li, Tao Sun

**Affiliations:** 1 Electronic Information School, Wuhan University, Wuhan 430072, China; xiaoliangwang@whu.edu.cn (X.W.); suntao@whu.edu.cn (T.S.); 2 GNSS Research Center, Wuhan University, Wuhan 430072, China; jiangp@whu.edu.cn; 3 Collaborative Innovation Center of Geospatial Technology, 129 Luoyu Road, Wuhan 430072, China

**Keywords:** Catmull-Rom curves, continuous-curvature, curvature upper bound, local regulation, path planning, unmanned aerial vehicles

## Abstract

Unmanned Aerial Vehicles (UAVs) play an important role in applications such as data collection and target reconnaissance. An accurate and optimal path can effectively increase the mission success rate in the case of small UAVs. Although path planning for UAVs is similar to that for traditional mobile robots, the special kinematic characteristics of UAVs (such as their minimum turning radius) have not been taken into account in previous studies. In this paper, we propose a locally-adjustable, continuous-curvature, bounded path-planning algorithm for fixed-wing UAVs. To deal with the curvature discontinuity problem, an optimal interpolation algorithm and a key-point shift algorithm are proposed based on the derivation of a curvature continuity condition. To meet the upper bound for curvature and to render the curvature extrema controllable, a local replanning scheme is designed by combining arcs and Bezier curves with monotonic curvature. In particular, a path transition mechanism is built for the replanning phase using minimum curvature circles for a planning philosophy. Numerical results demonstrate that the analytical planning algorithm can effectively generate continuous-curvature paths, while satisfying the curvature upper bound constraint and allowing UAVs to pass through all predefined waypoints in the desired mission region.

## 1. Introduction

Due to recent advances in communication, sensing and battery technologies, Unmanned Aerial Vehicles (UAVs) have drawn much attention for both military and civilian applications. Due to their portability and flexibility, UAVs are employed for numerous civilian applications, including industrial monitoring, scientific data collection and public safety (search and rescue). Soon, autonomous aircraft equipped with various sensors will be routinely surveilling our cities, neighborhoods and rural areas. They promise far reaching benefits for the study and understanding of public collaboration systems.

There are two main categories of UAVs: fixed-wing aircraft and multi-rotor vehicles. Compared with multi-rotor vehicles, fixed-wing aircraft are more advanced in many respects: they tend to be more stable in the air in the face of both piloting and technical errors as they have natural gliding capabilities even without power, and they are able to travel longer distances on less power. More importantly, they have the advantages of being able to fly at high speeds for a long time using a simpler structure. These characteristics make fixed-wing vehicles still widely popular, despite requiring a runway or launcher for takeoff and being unable to hover. For these reasons, we consider the path planning problem for fixed-wing UAVs in this work.

An important technical problem must, however, be addressed: How can we plan feasible and optimal paths that are effective are increasing the mission success rates and reducing the time taken? Path planning aims to determine a path from the initial position to the final position through a complicated space with or without obstacles. This is still an open problem in the field of autonomous systems research as it involves physical constraints due to the UAVs themselves and constraints due to their operating environment, as well as other operational requirements.

Several quintessential methods have been developed for path planning such as Voronoi diagrams [1], triangular-cell-based maps [2], as well as related grid-like maps [3,4], the potential function method [5], the A* algorithm [6,7], probability roadmaps [8], evolutionary algorithms [9,10,11,12] and mixed integer-linear programming [13,14]. Although these methods can provide optimal or near-optimal paths that pass through all predefined waypoints, they cannot guarantee smoothness, i.e., curvature continuity. Curvature discontinuities would cause sudden changes in the vehicle’s heading and threaten its safety. To generate suitable paths for UAVs, several problems need to be tackled.

(1) Path curvature continuity: This directly influences the vehicles’ aerodynamics and kinematics.
(2) Path optimization: We want to find the shortest path that passes through all of the expected waypoints. This is especially critical for missions such as reconnaissance and aerial delivery where vehicles are expected to fly over the specified target precisely, as any deviation would cause target information lost during reconnaissance and drop failures in transportation tasks.
(3) Local path adjustment: Dynamic environments require path replanning, which would consequently consume additional time. Accordingly, the planning method should be able to support local modifications.

The construction of feasible paths has been actively investigated in robotic research fields, because non-smooth motions can cause wheel slippage and degrade the robots’ dead-reckoning abilities [15]. Dubins curves are one practical method [16,17,18], as they can give the shortest path between given initial and final points with a fixed change rate of turn [16]. In these algorithms, two points are connected by concatenating arcs and tangents. Sahingoz et al. [18] and Shanmugavel et al. [19] apply Dubins curves to UAV path planning in 2D space after task allocation. Dubins curves have been extended to include other spline curves such as helix to generate more complex vehicle trajectory models in 3D space [17,20]. However, there are still curvature discontinuities at the junctions between lines and arcs, leading to yaw angle errors for UAVs. As a result, the corresponding applications are limited to straight flights.

To deal with the curvature discontinuity problem, two categories of curves are utilized in [21,22,23,24,25,26]. The first category involves parameterizing the curvature by arc length using, for example, clothoids [21,22]. Clothoid pairs can provide the shortest curves for a given turn angle [27]. However, there is no closed-form expression for position along the path or its approximation. Moreover, look-up tables are usually required, increasing the operational complexity. The second category can provide closed-form solutions, using, for example, B-splines, Bezier curves or spatial Pythagorean hodographs [23,24,25,27,28,29,30]. These curves are used for trajectory generation for both UAVs [23,24] and autonomous robots [25,28]. However, path curvature is a high-order function of curve parameters, and it is a formidable challenge to analyze its monotonicity. Generated paths have continuous curvature when using Bezier curves [31] or B-splines [32], but little attention has been given to the maximum curvature constraint. Although it has been demonstrated that curvature extrema can be obtained numerically for any planar cubic curve, this might be a challenge for parametric polynomial curves [29,33], since it is difficult to solve the formulated curvature extremum model. An objective function has been designed to search for proper Bezier curve parameters that satisfy the maximum curvature constraint, but the optimization problem remains unsolved [34]. Additionally, combined Bezier curves can be used to generate continuous curvature paths with bounded curvature extrema in both 2D and 3D space [24]. Shanmugavel et al. [30] have proposed a Pythagorean hodograph curve for 3D path planning, achieving bounded curvature and equal path lengths. Unfortunately, the generated path only passes through some of the waypoints, accordingly leading to failure of some specified tasks.

Despite all of these efforts, the challenge of generating feasible and optimal trajectories has been partially solved in the literature above. Furthermore, local path adjustment has been ignored, which is important in real applications. Replanning the whole path would be inefficient due to additional time and energy required. By contrast, local adjustment can reduce replanning complexity because the adjustment is used only where needed. Parametric curves with local adjustment, including B-splines and Beta-splines, can potentially be used for path planning. In particular, the construction of geometric continuous Beta-splines has been achieved by DeRose T D et al. In our work, the path planning problem is solved using a three-stage decomposition, which allows curvature continuity and curvature adjustment to be completely independent of each other. This strategy was inspired by the approach in [35], where circular orbits and Bezier curves are concatenated to create more complicated paths.

The objective of this paper is to develop path planning strategies that explicitly account for curvature and waypoint constraints. Previous work has primarily addressed path feasibility, whereas in this paper, we also consider path accuracy. In this paper, a continuous-curvature and bounded path planning algorithm for fixed-wing UAVs is proposed to solve the problem of passing through all of the waypoints under aerodynamic constraints. To this end, a parametric Catmull–Rom curve is employed.

The specific contributions of this paper are given below.

(1) A path planning framework is proposed to generate a feasible path that satisfies both curvature continuity and upper bound constraints.
(2) Path accuracy, which is absolutely necessary for aerial photography and air drop, is explicitly satisfied using the path planning algorithm.
(3) Geometric shaping of the replanned path can be systematically performed with the designed minimum curvature circle transition mechanism.
(4) The proposed path planning algorithm can also be able to solve obstacle avoidance problems to some extent.

The rest of this paper is organized as follows. Section 2 presents the problem formulation. In Section 3, a three-stage trajectory architecture is presented. In Section 4, a cross-value-based interpolation algorithm and a key-point shift algorithm are elaborated and the planar transition algorithm introduced. In Section 5, a path replanning algorithm using minimum curvature circle transitions is proposed. Simulation and experimental results are shown in Section 6, followed by the conclusion in Section 7.

## 2. Problem Statement

Let $WP=\left\{W_{1},\cdots ,W_{n}\right\}=\left\{\left(x_{1},y_{1}\right),\cdots ,\left(x_{n},y_{n}\right)\right\}$ be a sequence of waypoints in a 2D space returned by a higher planner. The path planning problem is to find a mission flight trajectory that fits all of the waypoints smoothly, satisfying curvature continuity and maximum curvature constraints.

For a feasible path curve, the curvature continuity and maximum curvature requirements must be satisfied simultaneously. Curvature discontinuity would lead to infinite changes in lateral acceleration, which can obviously not be allowed. Likewise, the curvature is proportional to the centripetal force at fixed velocity, and thus, unbounded curvature could result in roll angles that exceed the safety limits.

The smoothness of curve junctions can be measured by geometric and parametric continuity. Different orders of smoothness in terms of geometric and parametric continuity are defined in [36].

Parametric continuity: Let P($s_{0}$,$s_{1}$:s) and Q($t_{0}$,$t_{1}$:t) be parametric curves, such that $P\left(s_{1}\right)=Q\left(t_{0}\right)=m$. They meet at *m* with *k*-th order parametric continuity if:

(1) $$\frac{d^{k}P}{ds^{k}}=\frac{d^{k}Q}{dt^{k}},\;k=1,\cdots ,n$$

Geometric continuity: Let P($s_{0}$,$s_{1}$:s) and Q($t_{0}$,$t_{1}$:t) be parametric curves, such that $P\left(s_{1}\right)=Q\left(t_{0}\right)=m$. They meet at *m* with *k*-th order geometric continuity if the natural parameterizations of *P* and *Q* meet at *m* with parametric continuity.

These definitions are also used in this paper. Barsky et al. [36] point out that parametric continuity prevents parameterizations from generating geometrically smooth curves due to the strict constraints imposed by the derivatives. By contrast, geometric continuity accommodates differences between the parameterizations of adjoining curves, and thus, it is used to smooth the piecewise path in our work.

The UAV navigation limitations are due to its own kinematic constraints. As it has been widely proven that vertical motions consume additional energy, we also assume that the UAV is flying at constant altitude. The aerodynamics features of a fixed-wing UAV include lift force *L* and air friction *D*.

(2) $$L=\frac{1}{2}\rho V^{2}SC_{L}$$

where ρ is the air density, *V* is the flying speed, *S* is the wing area and $C_{L}$ represents the lift coefficient.

(3) $$D=\frac{1}{2}\rho V^{2}SC_{D}$$

where ρ is the air density, *V* is the flying speed, *S* is the maximum section area of an UAV and $C_{D}$ represents the resistance coefficient.

From the above two equations, we can see that the flying speed directly determines the lift force and air friction for a UAV under stable atmospheric conditions. In parallel, acceleration is generated from the corresponding component force, and this in turn changes the velocity of a UAV. Due to the designs themselves, fixed-wing UAVs have a minimum speed (the stall speed $V_{min}$), below which they risk falling out of the sky. In addition, UAVs are limited to a maximum speed of $V_{max}$ by power constraints. In practice, parameters including air density, wing area, lift coefficient, the maximum section area and the resistance coefficient were fixed. Thus, the dynamic constraint is the velocity: $\upsilon_{min}\leq \upsilon \leq \upsilon_{max}$.

To acquire the relation between curvature, velocity and lateral acceleration, a typical circular motion model is used to illustrate the turning motion for fixed-wing UAVs.

(4) $$F=\frac{mv^{2}}{R}$$

where *F* represents the centripetal force, *v* is the UAV’s velocity and *R* denotes the turning radius. Equation (4) can be further rewritten as: $\left|\kappa \right|=\frac{F}{m}=\frac{v^{2}}{R}$.

Since the curvature is inversely proportional to the turning radius *R*, the relation among curvature, velocity and lateral can be described as follows:

(5) $$\left|\alpha \right|={\left|v\right|}^{2}\kappa \propto \kappa$$

Due to the total centripetal force, which keeps constant during the turning, the curvature gets its minimum value ($\kappa_{min}$) when the flying speed reaches the extreme value ($v_{max}$). Consequently, the relation between maximum curvature, maximum velocity and lateral acceleration is:

(6) $$\left|\alpha \right|={\left|v_{max}\right|}^{2}\kappa_{min}$$

As far as the dynamics is concerned, (6) shows that the curvature is directly proportional to the vehicle’s lateral acceleration. The maximum curvature ($\kappa_{max}$) is inversely proportional to the minimum curvature radius ($\rho_{min}$) of the curves that it is able to follow. A path is valid if the curvature is smaller than the specified maximum value,

(7) $$\left|\kappa (t)\right|\leq \kappa_{max}$$

where $\kappa_{max}$ denotes the maximum curvature determined by the UAV’s dynamic constraints.

The path planning problem in this paper is independent of the UAVs’ dynamic models. The single or double integrator model is commonly used to characterize an individual UAV for state analysis. Since the power for a fixed-wing UAV is supplied by motors and direct user interactions are operations on the motor speed that change the state of a UAV, consequently, it is difficult to control the velocity and acceleration directly in engineering. For fixed-wing UAVs, which have a six degree of freedom movement, translational and rotational motions are achieved by adjusting propeller speed and the servomotor controller. Although different dynamic models are employed, the aerodynamic constraints are identical. To be specific, the curvature must be continuous and bounded in the 2D environment.

## 3. Three-Stage Trajectory Architecture

**Figure 1:**
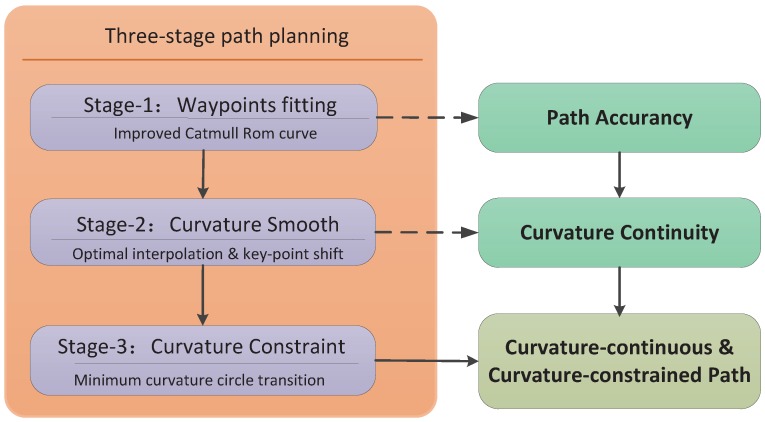
Workflow of the continuous-curvature path planning algorithm.

To deal with problems involving planning, a three-stage path planning algorithm with a transition selection mechanism for piecewise paths is proposed in this paper. The operational workflow of this algorithm is presented in Figure 1.

Our algorithm has two key components: curvature smoothing and curvature constraining. The Catmull-Rom curve is advanced in its local-control ability and owns the property that it passed through the control points. Thus, a Catmull-Rom curve is used to fit the waypoints firstly, to achieve path accuracy. Then, an optimal position or key point is extrapolated to smooth the curvature on the base of Phase 1. After that, to ensure that the UAV flies over each waypoint obeying the aerodynamic constraints, a minimum curvature circle transition scheme is used to replan those piecewise curves whose curvature exceeds the upper boundary.

Obstacle avoidance increases complexity for path planning and leads to path replanning. For off-line path planning, path curves should be designed to avoid obstacles using their prior knowledge such as positions and shapes. This results in constraints on path curves and consequently increases the algorithm complexity of path planning. As for on-line path planning, new paths must be planned rapidly to avoid dynamic obstacles, meaning that the whole path or local path should be replanned.

Our method was advanced in the organic coordination of path planning and obstacle avoidance. The method proposed can potentially solve some obstacle avoidance problems, while generating a continuous curvature and bounded path that passes through all waypoints.

**Figure 2:**
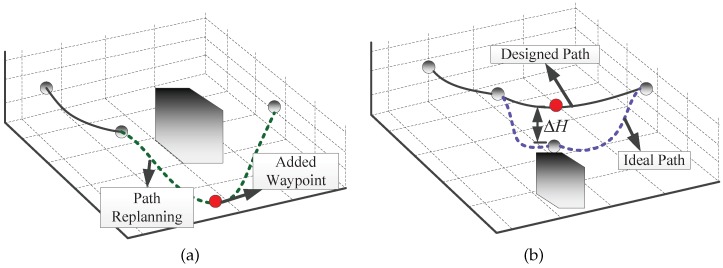
Two typical obstacle scenarios: (**a**) Obstacles are located between two waypoints (**b**) Obstacles are located below a waypoint.

In fact, the obstacle avoidance mechanism in Figure 2 is to navigate around obstacles by designing a path via path re-planning. By adding or moving a waypoint, the predefined path is locally changed. This path re-planning helps to solve the obstacle avoidance problem at the path level.

Specific to multiple waypoint path planning, there are mainly two types of obstacles as shown in Figure 2.

In the first scenario, we believe the collision avoidance problem can be solved by our proposed optimal interpolation method as demonstrated in Figure 2a. By adding a new waypoint, the path is replanned, and UAVs navigate around obstacles successfully. The research background of this paper is mainly ground observation using fixed wings UAVs where aeronautics and astronautic sensors are equipped on the vehicles for surface and human activities explorations. Under such background, the obstacle avoidance problem shown in Figure 2b can be tackled by driving the UAVs to fly over some waypoints. Although this operation results in range deviation between the flight path and waypoint and consequently reduces image quality, we believe that this quality decrease is allowable compared with the UAVs’ safety. Additionally, a PTZ (pan/tile/zoom) camera can also help to eliminate or mitigate the above negative influence.

## 4. $G^{2}$ Continuous Path Smoothing with Multiple Waypoints

It has been proven that Beta-constraints are necessary and sufficient conditions for $G^{n}$ continuity [37]. As an example, the Beta-constraints for $G^{2}$ continuity take the following form:

(8) $$\begin{array}{c} r^{\left(1\right)}\left(0\right)=\beta_{1}q^{\left(1\right)}\left(1\right)\\ r^{\left(2\right)}\left(0\right)=\beta_{1}^{2}q^{\left(2\right)}\left(1\right)+\beta_{2}q^{\left(1\right)}\left(1\right) \end{array}$$

where *r* and *q* are abbreviations for the parametric curves r(s0,s1:s) and q(t0,t1:t) and $\beta_{1}$ and $\beta_{2}$ are real numbers to represent the linear relationship between the deviation of parameter curves ($\beta_{2}$ can take arbitrary values, but $\beta_{1}$ must be positive).

Our goal has been to provide a path curve that passes through all waypoints and simultaneously satisfies Beta-constraints. It would be elegant to obtain an algebraic solution demonstrating the curve’s adequacy and completeness, but it is arduous to solve sets of second order differential equations with two degrees of freedom since it is formidably difficult to find closed-form solutions. We instead present a practical algorithm based on the idea that a geometric method can be used to design a quantic interpolating spline, which is a subclass of the Catmull–Rom splines with two shape control parameters [38]. In this section, a Catmull–Rom-based continuous-curvature condition is derived, and a relevant path smoothing algorithm is presented.

### 4.1. Curvature Continuity Condition

In order to get a better and clearer understanding of the work below, the construction method of the Catmull–Rom curve is briefly introduced in this section. The method consists of three parts: set shape parameters, calculate Bezier polygon, determine Bezier control points. The effect of shape parameters was studied in [38], and they were recommended to be set as $\beta_{1}=1$ and $\beta_{2}=0$. After iteration calculation, the polygon of an auxiliary Bezier curve was figured out. Finally, the expected Bezier control points were determined by the linear weight of the auxiliary Bezier polygon.

**Figure 3:**
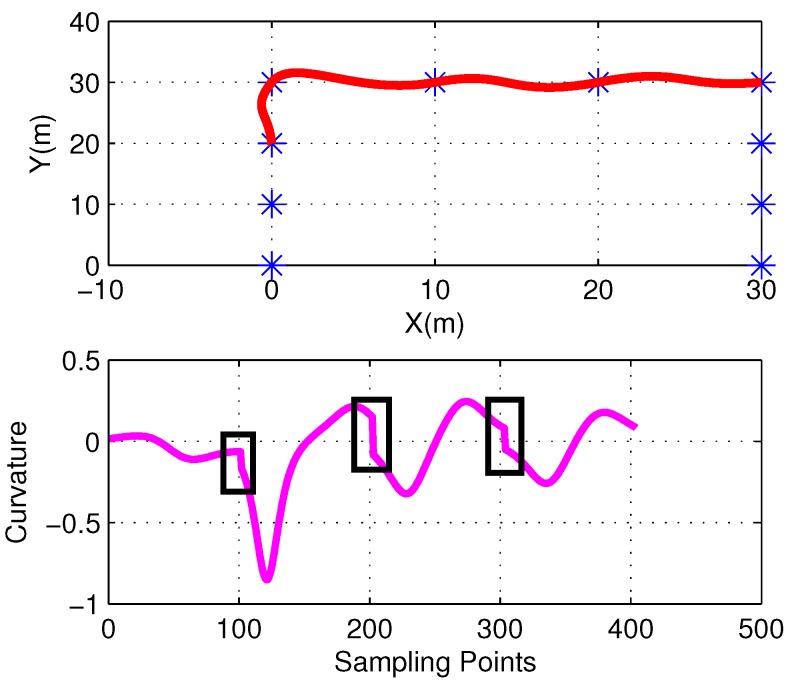
Discontinuity of the curvature value at the junction points: The red line is original path curve and the purple one represents its curvature value.

For predefined waypoints, curvature discontinuities may exist at junction points, as shown in Figure 3. Further investigation indicates that the shape parameters and positions of the corresponding waypoints are the decisive factors in determining the degree of discontinuity. For simplicity’s sake, we set the shape parameters as follows: $\beta_{1}=1$ and $\beta_{2}=0$. It should be noted that although the results in Equation (9) would change when the parameters are different, the curvature smoothing algorithm proposed still holds.

According to the Catmull–Rom curve construction method given in [38], the curvature change at the point $\left(x_{i},y_{i}\right)$ is given by:

$$\Delta \kappa =(x_{i–2}y_{i}–x_{i}y_{i–2}–9x_{i–1}y_{i}+9x_{i}y_{i–1}–7x_{i}y_{i+1}\cdots$$

(9) $$\cdots +7x_{i+1}y_{i}+x_{i}y_{i+2}–x_{i+2}y_{i})/900\;$$

To ensure curvature continuity, the above condition is used to calculate point position in optimal interpolation and key-point shift algorithms.

### 4.2. Path Smoothing Algorithm

The curvature continuity condition essentially puts constraints on the relative positions of waypoints. If the local distribution of the original points is properly adjusted, curvature continuity can be achieved. Although (9) is the strict mathematical condition for curvature continuity, this theoretical requirement is difficult to satisfy in practice due to project errors such as measurement or mechanical errors. Thus, we regard the curve as continuous as long as the curvature change is less than a given limit ε. Various tasks have different requests with respect to time cost. For example, a loose time constraint is needed in aerial photography tasks. By contrast, missions such as rescue and reconnaissance require that the time taken must be as little as possible. To meet different demands on time cost while satisfying the continuous-curvature condition, two algorithms (optimal interpolation and key-point shift algorithm) were proposed.

**Figure 4:**
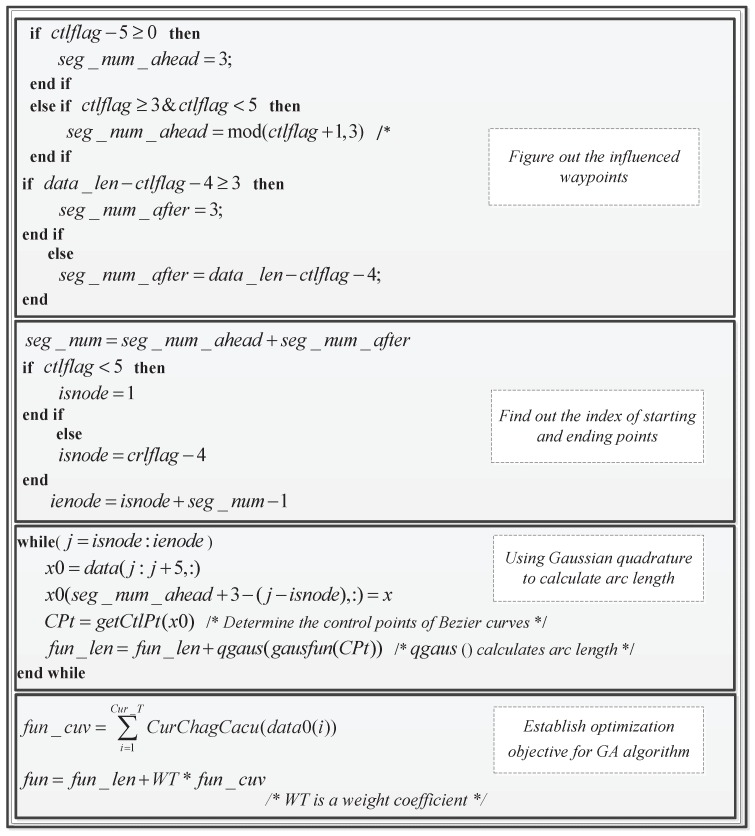
Description of the optimal interpolation algorithm.

The core idea of the optimal interpolation algorithm is to find a reasonable position to insert a new waypoint. Equation (9) shows that a new waypoint would influence the curvature of five neighboring junctions. Consequently, the goal is to find a point that makes the associated curvature changes tend to zero. By considering how the control points of nearby piecewise curves are affected by the inserted waypoint, the optimization objective is to minimize the length of these curves. As a result, the proposed algorithm solves the following optimization problem.

$$\min f(x;y)=\sum_{1}^{10}\ell_{j}$$

(10) $$s.t.\left\{\begin{array}{c} \left|\Delta \kappa_{i}\right|\leq \varepsilon \;\left(i=1,2,\cdots ,5\right)\\ x\in X\\ y\in Y \end{array}\right.$$

where $l_{i}$ represents the related path length and ε denotes a user-defined continuity threshold. Numerical methods such as steepest descent and Newton’s method are usually employed to solve such optimization problems, but in practice, the integral for calculating the arc length cannot be solved. By contrast, the Gaussian quadrature method only needs the polynomial form of the integral and selects the expected number of points on the integral interval, reducing the computational complexity dramatically. To increase the computational efficiency, both the Gaussian quadrature method and a Genetic Algorithm (GA) are adopted. The input and output of the algorithm are the set of waypoints and the coordinates of the objective point, respectively.The main steps of the optimal interpolation are described in Figure 4.

The optimal interpolation algorithm mainly consists of two phases: construct a cost function and solve the optimization problem. Since the task cycle is a key factor that determines the optimization of flight paths, the path length has also been taken into consideration in the cost function. To obtain the explicit formulation of the cost involved, numerical computation is conducted via Gaussian Quadrature (GQ). Considering that the lengths of adjacent piecewise paths are effected by the newly-added waypoint, those related sequence numbers of waypoints are figured out firstly. Then, the length of each segment is estimated by GQ. As for the curvature change value, the result from Equation (9) is used.

The GA algorithm that was used to solve the optimal interpolation problem was a function encapsulated in a toolbox by MATLAB. The function is: *X = ga(fitnessfcn,nvars,A,b,Aeq,beq,LB,UB)*. In the GA algorithm, the input is curve length and curvature change values with corresponding weight coefficients, and the output is the coordinate of the objective point. Some key parameters in the optimal interpolation algorithm are given as follows: ctlflag is the sequence number of the point needed to be interpolated; Seg_Num is the total number of piecewise curve; seg_num_ahead represents the number of segments that are located ahead of the interpolated point; seg_num_after denotes the number of segments that are located behind the interpolated point; isnode,ienode are the start and end sequence number of the points affected by the interpolation operations, respectively; data_len is the length of the new waypoint set; fun_len is the total path length related to the interpolation point; CPt are the control point of Bezier curves; and fun_cuv is the total curvature change value.

The key idea of the key-point shift algorithm is to displace as few waypoints as possible and then replan the trajectory using these new points. Due to curve shape changes, the two proposed algorithms both result in path length increment. Thus, curvature smoothing and curve length are two main factors considered in path smoothing. Consequently, we set the curvature change value as the constraint and the curve length as a target, as shown in Equation (10), in both the optimal interpolation and key-point shift algorithms. The optimization problem can be formulated as in Equation (10).

To solve the above optimization problem, weight coefficients ($w_{1}$, $w_{2}$) were set for the curvature change value and path length, respectively, in the optimal interpolation and key-point shift algorithms.

However, curvature continuity is achieved through curve shape changes by the added piecewise path in the optimal interpolation algorithm, while the key-point shift algorithm is to displace as few waypoints as possible, avoiding the introduction of new waypoints. This results in a much greater increment in path length for the optimal interpolation algorithm. To obtain satisfactory results, different weight coefficient were assigned ($w_{1}$ = 800−3000, $w_{2}$ = 500−3000) in the optimal interpolation algorithm and ($w_{1}$ = 800−3000, $w_{2}$ = 100−500) in the key point shift algorithm. The results of $\omega_{1}$ and $\omega_{2}$ are empirical values based on a large number of numerical experiments.

## 5. Minimum Curvature Circle Transition

To ensure path feasibility for a given UAV, the curve ($\vec{s(t)}$) must fulfill certain aerodynamic constraints. The maximum curvature ($\kappa_{max}$) is an achievable constraint by the vehicle in 2D space. Although the path generated in Section IV has continuous curvature, the curvature extrema have yet to be considered. Those piecewise paths whose curvatures exceed a specified threshold are physically unreachable. Here, a geometric path transition mechanism is established to meet these curvature upper bound constraints.

**Figure 5:**
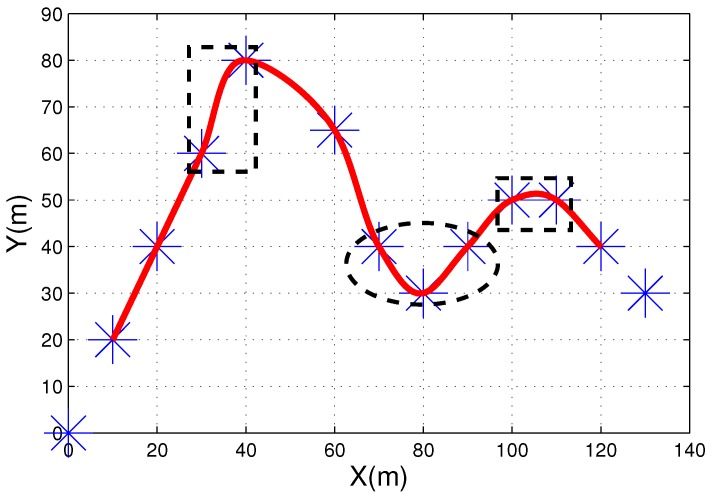
No waypoint on path and one waypoint on path cases: The piecewise curves are shown within the rectangular and elliptical boxes.

Considering the complete space of solutions, two classes of over-curved path transition are proposed: no waypoint on path and one waypoint on path. When replanning a piecewise path, it is essential to understand what kind of transition problem it presents so that it can be solved in an appropriate manner. Figure 5 shows the two classes of path transition. The first category (shown in the rectangular boxes) is where the over-curved segment is a part of the path curve between two adjacent waypoints. Similarly, the second category (shown in the elliptical boxes) represents curve segments that pass through a waypoint.

### 5.1. Analytical Solutions of the Minimum Curvature Circle Transition

It has been demonstrated that a cubic curve can be used for transitions preserving $G^{2}$ continuity and that two variables (m,θ) are required to generate the expected connections [35]. From the results of [35], it appears that the value of parameter *m* was determined by trial and error. Here, we want to determine the value of *m* for given star and end point. This can be achieved using the following theorem.

**Theorem** **1.** *If the centers of the two minimum curvature circles are known, there is a unique curvature-constrained curve connecting two points, one located on each circle.*

**Proof.**  When a local frame is set up, the angle between the x-axis and the line segment between the two circle centers is (for *s*-shape):

(11) $$\alpha =\arctan\frac{16m^{2}\cdot \frac{\sin^{2}\theta }{27\cos^{2}\theta }\cdot r_{0}^{2}}{2\cdot Pr_{0}\cdot \sqrt{\frac{2\sin\theta }{3}}+h\cos\theta }$$

The parameters for *s*-shaped and *c*-shaped curves can then be obtained from (12) and (13), respectively.

(12) $$\left\{\begin{array}{c} \left(8r_{0}\alpha \varphi –8r_{0}\varphi^{2}\right)m^{2}+24r_{0}\alpha \varphi m+54r_{0}=0\\ 8r_{0}\varphi^{2}m^{2}–\left(2r_{0}\left(9–12m–2m^{2}\right)+\delta \right)=0 \end{array}\right.$$

$$\delta =\sqrt{729r^{2}+{r_{0}}^{2}{\left(6+2m\right)}^{2}\left(4m^{2}+24m–72\right)}$$

(13) $$\left\{\begin{array}{c} 2{r_{0}m}^{2}\varphi^{3}–3x_{1}\varphi^{2}+\left(\Delta –3r_{0}\right)\varphi –3x_{1}=0\\ 2{r_{0}m}^{2}\varphi^{4}+\left(\Delta –3y_{1}\right)\varphi^{2}+3r_{0}–3y_{1}=0 \end{array}\right.$$

This is derived by assigning $P=m\cdot \frac{1}{\cos\theta }\cdot \sqrt{\frac{8\sin\theta }{27}}$ and $h=m^{2}\cdot \frac{1}{\cos^{2}\theta }\cdot \frac{8\sin\theta }{27}\cdot R_{0}$ to (12) and (13).

Here, Δ is equal to $2r_{0}m^{2}+4mr_{0}–3r_{0}$.

$r_{0}$ is the radius of the minimum curvature circle; φ is equal to $tan\left(\theta \right)$; α is the angle tangent of the x-axis and the straight line connecting two circle centers; *r* represents the distance between the circles’ centers; and $\left(x_{1},y_{1}\right)$ denotes the center coordinate where endpoints are located. ☐

It can be seen that a feasible transition can be figured out by solving (*m*,φ) from Equation (13).

### 5.2. Transition Model for the No Waypoint on Path Case

**Figure 6:**
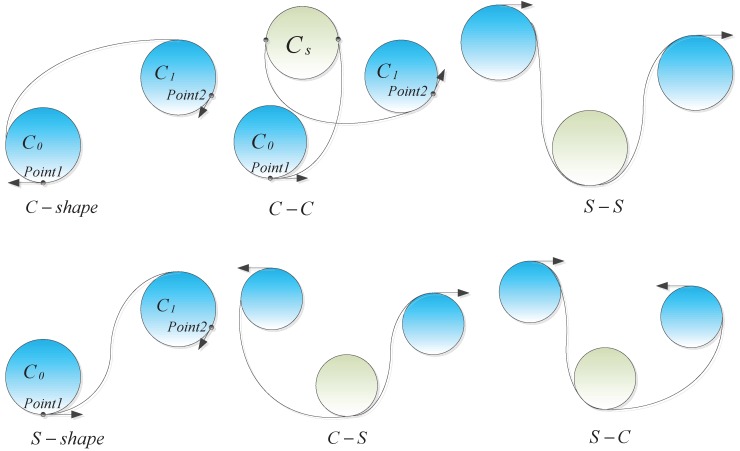
Transition instances: *s*-shape, c–s and s–c transitions reverse the tangent direction, while *c*-shape, c–c and s–s transitions reserve this direction.

Inspired by the approach taken in [35], where curvature-bounded connections are achieved by utilizing arcs and cubic Bezier curves, we extend the basic *c* and *s*-shaped curves to develop a series of hybrid transitions, which include c–c, s–s, c–s and s–c shapes. Figure 6 shows an enumeration of these shapes, where we can see that both the shape and length of the curves vary substantially between transition types. Since UAVs proceed in a predetermined direction along their trajectories, the tangent to the transition curve must be considered. Transition curves can be divided into two categories depending on whether or not the rotation orientation switches between the start and end points. To be specific, the *c*, c–c and s–s shapes guarantee that the curve goes clockwise or anticlockwise, while it reverses direction for *s*, s–c and c–s shapes.

To satisfy the maximum curvature condition, a minimum-curvature circle is employed to pass through the points whose curvature values exceed the threshold. A minimum curvature circle is defined as one whose curvature radius is in accordance with the settled curvature threshold. As the minimum curvature circle transition model is built in a local coordinate system, the global coordinates have to be converted to the local coordinates. We set the first point with the curvature threshold as the center of the local coordinates. The transformation matrix is constructed as follows.

(14) $$\left[x\;y\right]=\left[x^{\prime }\;y^{\prime }\right]R+T\;$$

The transformation matrix includes rotation and translation operations, ***R*** and ***T***, respectively. Using the inverse of this matrix, the local coordinates can be derived from the global reference system:

(15) $$P_{g}=\left(P_{l}–T\right)\cdot R^{–1}\;$$

where $P_{g}$ denotes the global coordinates and $P_{l}$ denotes the local coordinates.

Although the points to be connected are located on the minimum curvature circles, it can be shown that both the *s* and *c*-shape transition formulas hold in a bounded interval, which is determined by Theorem 2.

**Theorem** **2.** *The lower bound on the feasible interval is determined by setting m to one and the upper bound by the intersection of the inner or outer tangent lines and circles, respectively, for s and c-shape transitions.*

**Proof.**  We consider an *s*-shape transition curve Z(t) here. Set the angle of $\vec{P_{2}P_{3}}$ and $\vec{P_{1}P_{2}}$ to θ. In addition, $\vec{P_{1}P_{2}}$ and $\vec{P_{2}P_{3}}$ are parallel to the x-axis. This can be summarized as:

$$P_{0}=\left(0,0\right)\;P_{1}=\left(g,0\right)\;P_{2}=\left(g+h\cos\theta ,h\sin\theta \right)$$

$$P_{3}=\left(g+h\cos\theta +k,h\sin\theta \right)$$

where *g*, *h* and *k* are the straight-line distances between the control points of Bezier curves. $P_{i}\left(i=0,1,2,3\right)$ are the control points of Bezier curves. According to the results in [34], whether or not the curvature derivative of Z(t) vanishes on [0,1] depends on the value of *Q*, where:

$$Q=3\cos^{2}\theta f_{1}\left(s\right)+4m^{2}sf_{2}\left(s\right)$$

We also have:

$$Q=Am^{2}+Bm+C$$

where:

$$\begin{array}{c} A=12s^{4}–4s^{3}–4s^{2}+12s,\\ B=–3\left(3s^{5}–5s^{4}+10s^{3}+10s^{2}–5s+3\right)\cos^{2}\theta ,\\ C=3\left(s^{5}–2s^{4}–s^{3}–s^{2}–2s+1\right)\cos^{2}\theta . \end{array}$$

Letting θ→0 yields $\lim_{m\to 1,s\to 0}Q=0$, while ∃s, which makes Q≤0 as m≤1. Meanwhile, the value of *h* approaches zero when the transition curve points start at the intersection of the inner tangent lines and the circle. ☐

From the above proof, we can see that θ→0 and h→0 determine the boundary condition for *s*-shape transition.

### 5.3. Approach for the One Waypoint on Path Case

In addition to the approaches above, a hybrid transition mechanism has also been proposed for more complicated situations. Although the *s*-shape and *c*-shape models can solve curve transition problems between two points with controllable curvature extrema, this mechanism is essentially for determining the parameters of the cubic Bezier curves’ control points using curvature monotonicity conditions. Compared with the situations studied in Section 5.2, a much more complicated application is one where one or more waypoints on the path need to be replanned. A single *s*-shape or *c*-shape solution could not satisfy replanning requirements because the transition curve control parameters are determined once the start and end points are given. In such cases, the curve shapes are fixed, and thus, the requirement to pass through the waypoints cannot be ensured. Therefore, changes are needed for each pair of points on the path curve. To solve this problem, a hybrid transition approach is proposed, which includes the following combined connections: s–s-, c–c-, c–s- and s–c-shaped curves. Using these, an auxiliary minimum curvature circle can be constructed to reach the specified waypoint. Afterwards, an *s*-shape or *c*-shape transition is employed to connect the points to this circle within the curvature threshold via piecewise arcs.

The key to finding solutions for these hybrid transitions is to estimate an appropriate position for the center of a minimum curvature circle. Results in Section 5.2 prove that a transition is valid when the start and end control points of an *s* or *c* curve fall within restricted ranges. For this reason, there is a wide choice of possible curve parameters, which give rise to different curve lengths. To optimize the path, an optimal position for the center must be found.

**Figure 7:**
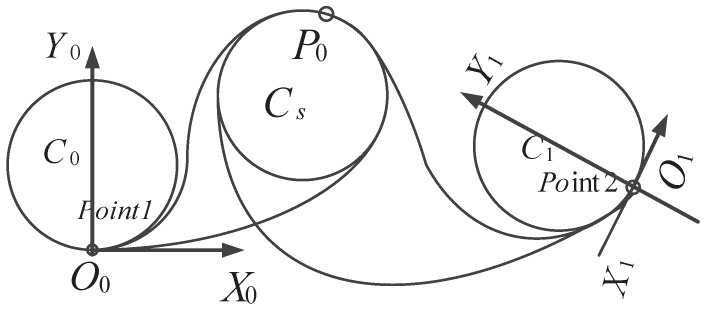
A minimum curvature circle transition example for the one waypoint on-line case.

To determine the optimal center position of the auxiliary circle, the boundary conditions should first be established. Results in Section 5.2 show that the points of tangency on the inner tangent lines between two minimum circles are upper bounds on the end points for *s*-shape transition curves. The lower bounds can be determined by setting *m* to one. As an illustrative example, consider three pairs of points and their minimum curvature circles, as shown in Figure 7. Here, Point 1 and Point 2 are the predetermined start and end points, and $P_{0}$ is a predefined waypoint. The tangent directions at the start and end points are both counter-clockwise. Additionally, the fixed point $P_{0}$ is required to be on the sub-path. For simplicity’s sake, the circle that passes through $P_{0}$ is defined as an auxiliary circle as follows.

**Figure 8:**
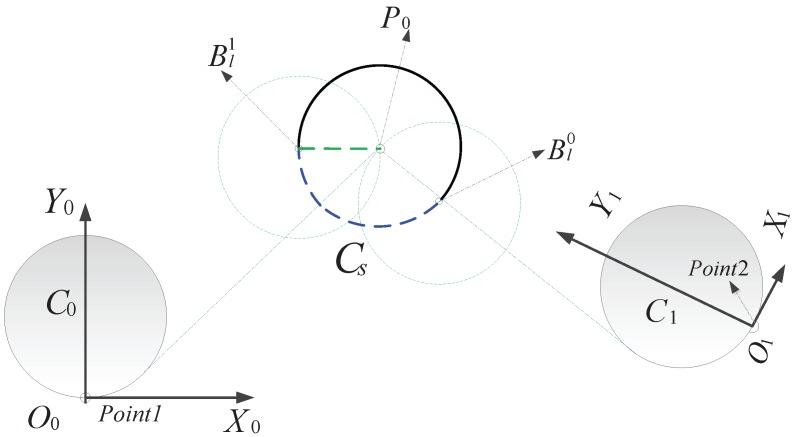
Circle center trajectory bounds for the s–s transition.

By constructing an outer tangent line to $C_{0}$ (the minimum curvature circle passing through Point1) from $P_{0}$ as shown in Figure 8, the intersection point determines a bond on $C_{s}$. This is because the end point of the transition curve crosses the waypoint as the circle center moves in a counter-clockwise direction along its orbit, and thus, we want the UAV to move along an arc on the minimum curvature circle to travel across the waypoint, leading to an increase in path length. In addition, another bound on $C_{s}$ can be obtained by drawing a line parallel to the x-axis from point $P_{0}$, which intersects the circle at $B_{l}^{1}$ as shown in Figure 8. As the circle center moves clockwise, the horizontal velocity component simultaneously reverses direction. Consequently, bounds on the circle center for an s–s transition are given by the blue dotted line. Auxiliary circle center bounds for other hybrid transitions can be acquired likewise.

Compared with conventional search algorithms, binary search has the advantages of low time complexity and no need for any other search region interventions. Thus, it is used to determine the position for the center of the auxiliary circle. The iterative process is summarized in Algorithm 1.

**Algorithm 1** Binary searching algorithm for optimal circle center.Input: Lower and upper bound Output: Optimal circle center 1: Initialize the circle center (*x*,*y*); 2: Determine the starting position $C_{s,s}$ and the ending position $C_{s,f}$ ; 3: Let $C_{s,s}$ and $C_{s,g}$ be the initial and final poses of the circle center trajectory, and compute the center ($x_{0}$,$y_{0}$) and ($x_{1}$,$y_{1}$); 4: **while**
$\left(\left(x,y\right)\in \left[C_{s,s}C_{s,f}\right]\right)$ ; 5:  **if**
$\left(\left|\sum_{i=0}^{2}\ell_{i}\left(x,y\right)–\sum_{i=0}^{2}\ell_{i}\left(x_{0},y_{0}\right)\right|>0\right)$
**then**; 6:   $\left(x,y\right)=(\left(x,y\right)+\left(x_{0},y_{0}\right))/2$ ; 7:  **else** 8:   **Break**; 9:  **end if** 10: **end while**

The above process consists of two phases: specification of the search region and iterative search. Utilizing the auxiliary circle center bounds determined by the previous analysis, we create an objective function that optimizes for curve length. As a result, the iteration terminates when the minimum length is obtained. Binary search is well known to have a time complexity of O(log(n)).

### 5.4. Novelty of the Proposed Transition Method

Although constructing curves with monotonic curvature using arcs and Bezier curves is quite standard in math, our method was unique as we apply the simple method in UAV path planning. The novelty is exhibited as follows: (1) on the condition that the re-planned curves had monotonic curvature, arrival of intermediate waypoints was achieved by using a minimum curvature circle; (2) s–s-, s–c-, c–c- and c–s-shaped curves were designed to connect adjacent waypoints, ensuring a UAV flied along the predefined direction; (3) the shortest replanning path was determined using the binary searching method on the basis of the auxiliary circle center’s boundary condition.

## 6. Experiments and Discussion

To evaluate the performance of the proposed algorithm, simulated experiments were carried out to generate continuous-curvature constrained paths. To demonstrate the superiority of the proposed algorithm, typical path planning methods including the $G^{2}$ Bezier curve and Dubins curves were compared with the proposed algorithms.

### 6.1. Continuous-Curvature Bounded Catmull-Rom Path Generation

To verify the effectiveness and robustness of the proposed method, the parameters of a physical fixed-wing UAV were used for the numerical simulations.

The parameters for each experiment are listed in Table 1. It should be noted that the turning acceleration, fixed as 3 g, was determined by the aircraft itself.

**Figure 9:**
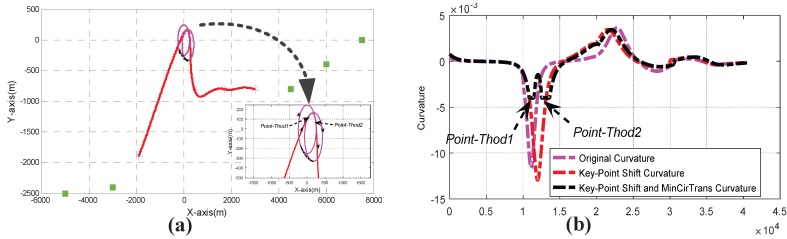
Path generation: (**a**) *c*-shaped transition (**b**) Curvature value of path curves.

**Figure 10:**
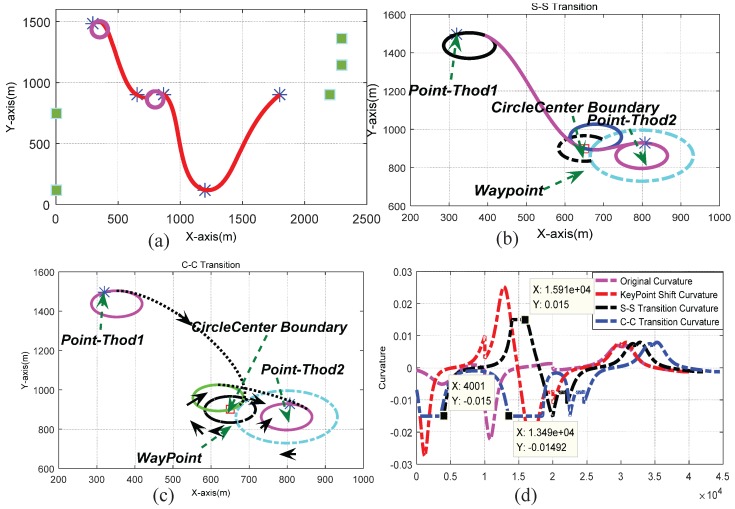
Combined transition mechanisms: (**a**) Final path (**b**) s–s transition (**c**) c–c transition (**d**) Curvature value of corresponding curves.

Firstly, the key-point shift algorithm incorporated with the *c*-shaped transition mechanism was used to generate a feasible path as shown in Figure 9a. The corresponding curvature of the path curve is as displayed in Figure 9b. From the results, we can see that the curvature of the designed path is continuous and under upper bound constraints. The hybrid transitions included the s–s, c–c, s–c and c–s cases. Figure 10 shows the results of using s–s and c–c transitions for local replanning. The s–c and c–s solutions were scrapped due to the tangent directions of the start and end waypoints. The limiting case for an s–s transition is that two circles are tangent to each other, while a c–c transition only requires the circle centers to be outside each other’s circumference. The center trajectories of the curvature circles for the intermediate waypoints are shown as black arcs in Figure 10b,c. Binary search was used to determine the appropriate circle center positions. Since transition paths are composed of circle arcs and Bezier curves with monotonic curvature, the curvatures of both the s–s and c–c transitions are bounded by the maximum value $\kappa_{max}$.

### 6.2. Path Smoothing Algorithm Comparison

**Figure 11:**
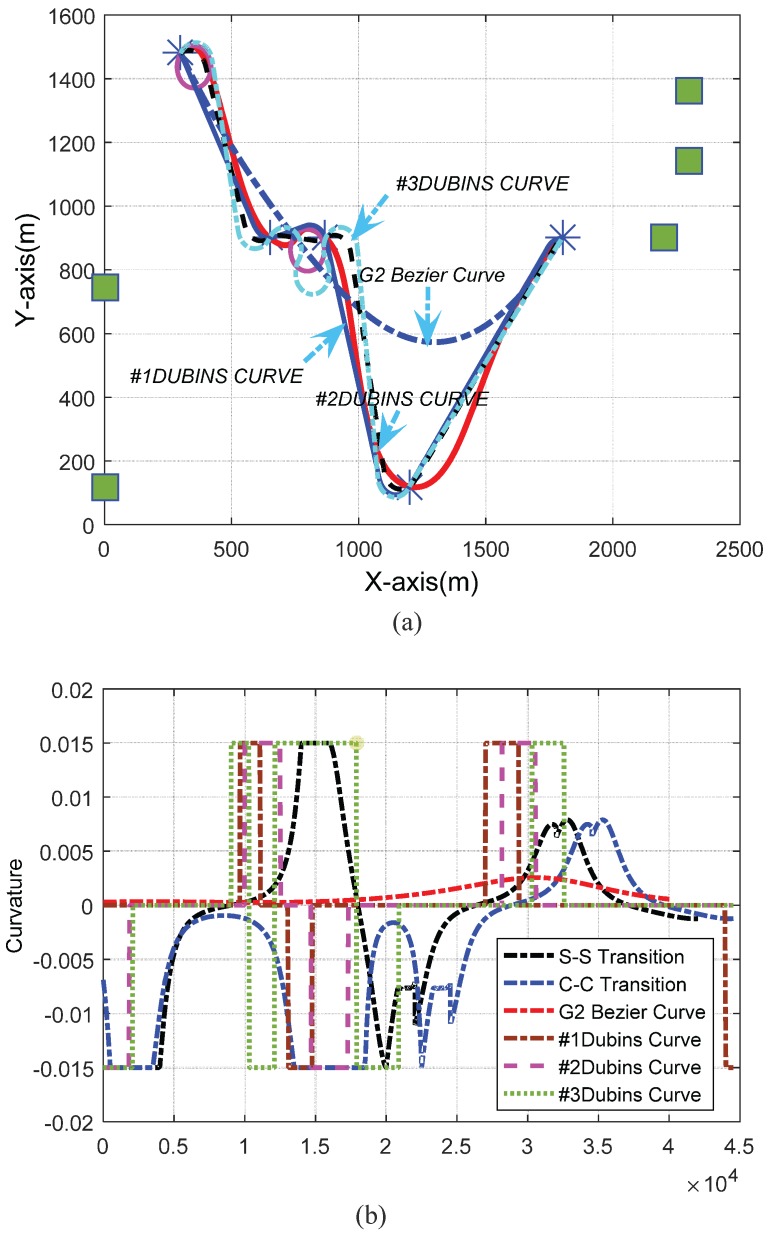
Performance Comparison of different path planning results: (**a**) Curve shape of $G^{2}$ Bezier, Dubins, s–s and c–c curves; (**b**) Comparisons of corresponding curvature value.

To evaluate the effectiveness of the proposed path planning method, we compared the performance of the three different curves: $G^{2}$ Catmull-Rom, Dubins, and $G^{2}$ Bezier. Figure 11a presents the predefined waypoints and paths generated by the three kinds of methods. Three Dubins curves with different steering angles are given in Table 2, while the path lengths of three algorithms are listed in Table 3. The shortest distances from the designed path to all of the control points are also given in Table 3 to compare the paths produced by the three methods. The path curvatures are shown in Figure 11b, where it can be seen that only the proposed method generated a path whose curvature was both continuous and bounded.

Based on the results given in Table 3 and Figure 11b, we can see that the $G^{2}$ Catmull-Rom method can generate a continuous curvature, locally-adjustable path. Although the curvature of the $G^{2}$ Bezier curve was continuous and under the specified limit, the path merely approximated the control polygon, meaning that it would have missed some control points. Additionally, the Dubins curve paths travel across all of the waypoints, but have discontinuous curvature values at the junction points. We employed quintic Bezier curves to connect all of the waypoints and developed a minimum curvature circle transition method to solve the over-curvature problem. Thus, both the curvature continuity constraint and the curvature upper bound were satisfied. Table 3 does, however, indicate that the designed path was longer than the $G^{2}$ Bezier curve path. This is mainly because approximating splines were adopted between sequential waypoints to achieve an interpolating curve as a whole result. Meanwhile, due to the tangent direction at each waypoint, a *c-c* transition needs circular arcs as sub-paths to adapt the flight direction, which leads to even longer paths than using *s-s* transitions.

Furthermore, we can see from Figure 9b and Figure 11b that the curvature extremes varied with the predefined maximum flying speed. This is because the curvature is inversely proportional to a UAV’s velocity with fixed lateral acceleration.

**Figure 12:**
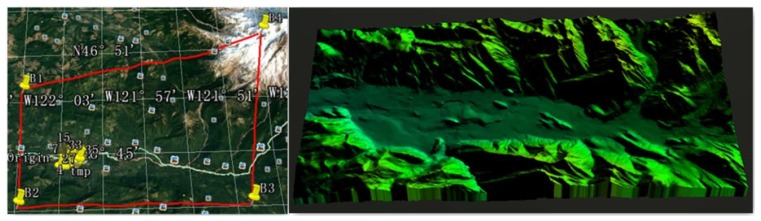
Areas in the experiments.

Further, we have also tested our methodology using fixed-wing UAV models in STK, which is a physics-based software package from Analytical Graphics, Inc (220 Valley Creek Blvd. Exton, PA USA). The turn acceleration (an inherent parameter of the UAV) is 29.42 m/s^2^, and the cruise velocity is fixed as 160 km/h. To simulate the real flying conditions, we chose an area from Google Earth, and for the verification of the algorithm, the elevation map of the same region obtained from GlobalMapper was used. The experiments were carried out by using a topology map as shown in Figure 12, the longitude of which ranges from $121^{\circ }$50’5.99” W–$122^{\circ }$5’52.79” W, and the latitude ranges from $46^{\circ }$42’3.6” N–$46^{\circ }$48’32.4” N.

**Figure 13:**
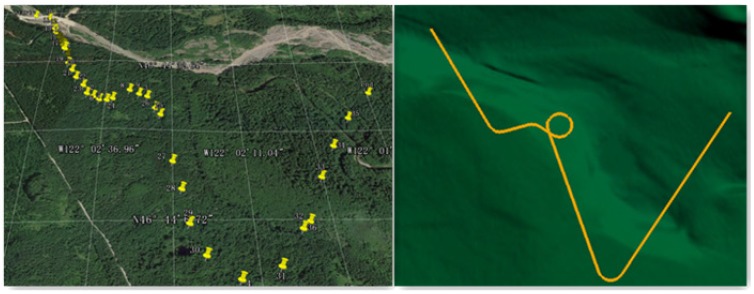
Pre-designed path and motion trajectory.

**Figure 14:**
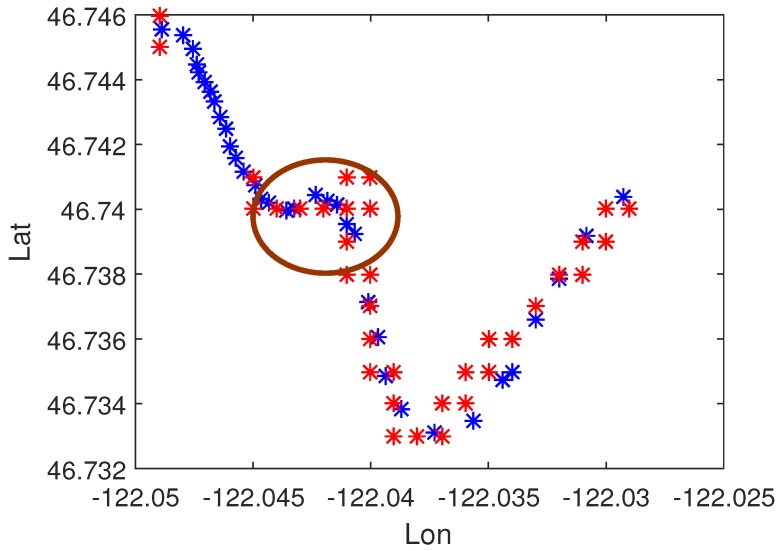
Comparisons between the designed and actual paths: the blue markers are designed path and the red ones represent the actual path.

In order to observe the effect of the generated path, the path was discretized into points that were embedded into the UAV control system. Figure 13 shows the path generated by the proposed algorithm and the realistic trajectory. The result comparisons are demonstrated in Figure 14. The blue markers represent the path generated by our method, while the red ones denote the realistic trajectory. From the comparisons, we can see that the motion trajectory of the UAV coincides with the trajectory of planning. A remarkable detail was shown circled in maroon. It can be seen that the pre-designed path had the shape of a bump, but the UAV actually flies along a circle. This is because an s-s-shaped transition was used in our method, while the UAV followed a c-c-shaped transition. In addition, we can also see the phenomenon of path deviation in some piecewise paths. However, these path biases were mainly caused by two reasons: resolution restriction on Google Earth and GPS positioning error. Google Earth provided DEM of an area with 0.5–1-m resolution, while the positioning error of commercial GPS can be 5–20 m. This consequently resulted in path errors under those allowed.

### 6.3. Discussion

To solve the UAV path planning and optimization problem, the following requirements must be satisfied: path feasibility and path accuracy. Paths generated by the proposed method and by using other curves, including Dubins and $G^{2}$ Bezier curves, were compared in terms of path feasibility and accuracy in Section 6. Figure 11b demonstrates that the curvatures of the paths designed by our method were continuous and bounded, indicating their superior path feasibility. From Figure 11a, we can see that the designed path passes through all of the predefined waypoints, demonstrating our method’s superiority in path accuracy.

## 7. Conclusions

In this paper, a continuous-curvature path planning algorithm was proposed, which allowed the generated path to pass through all of the sequential waypoints while satisfying the curvature upper bound constraint. A curvature continuity condition has been derived for parametric Catmull-Rom curves. Based on this condition, an optimal interpolation algorithm and a key-point shift algorithm were developed to achieve continuous curvature. Furthermore, a minimum curvature circle transition method was designed to replan the over-curved sub-paths using circular arcs and Bezier curves. In particular, a transition selection mechanism was established in accordance with the distribution of waypoint. Numerical results demonstrated that the algorithm was capable of generating a continuous path meeting the curvature upper bound constraint.

This work is just the beginning of the implementation of this approach in the navigation of UAVs during autonomous missions for ground observation. In future works, we will develop a path-following algorithm incorporate in our problems, and we will analyze the path under external disturbances (e.g., wind field). We are interested in extending our results to 3D environments.

## Figures and Tables

**Table 1:** Parameters for the numerical experiments.

Turn Acceleration (m· s^−2^)	*R* (m)	κ	$V_{\max}$ (m/s)
29.42	250	0.004	85.76
29.42	66.667	0.015	44.3

**Table 2:** Steering angles of the Dubins curves.

	φ1	φ2	φ3	φ4	φ5
#1 Dubins	−1.027	0	−1.17	0.915	0
#2 Dubins	0.5236	0.5236	0.5236	0.523	0
#3 Dubins	1.0472	1.0472	1.0472	1.0472	1.0472

**Table 3:** Comparison of the three types of methods.

	$G^{2}$ Bezier	#1 Dubins	#2 Dubins	#3 Dubins	s–s	c–c
$\left|\kappa_{max}\right|$	0.0025	0.015	0.015	0.015	0.015	0.015
Path Length	2012.9	2859.1	2933.5	3509.1	3483.5	2775.7
Point 1	0	0	0	0	0	0
Point 2	73	0	0	0	0	0
Point 3	85	0	0	0	0	0
Point 4	459	0	0	0	0	0
Point 5	0	0	0	0	0	0
